# Sensory nerve action potential in patients with functional neurological disorders with sensory manifestations

**DOI:** 10.3389/fneur.2023.1259887

**Published:** 2023-10-25

**Authors:** Kohei Morimoto, Kenji Sekiguchi, Riki Matsumoto

**Affiliations:** Division of Neurology, Kobe University Graduate School of Medicine, Kobe, Japan

**Keywords:** functional neurological disorder, sensory symptom, nerve conduction study, sensory nerve action potential (SNAP), FND

## Abstract

**Introduction:**

Functional neurological disorder (FND) has various clinical manifestations. Even though diagnostic criteria for FND have been proposed, FND characteristics with sensory manifestations have not been elucidated. Therefore, we aimed to investigate the association between sensory nerve action potential (SNAP) amplitudes and FND with sensory manifestations.

**Methods:**

We included 76 outpatients with FND with sensory manifestations whose nerve conduction studies were performed retrospectively. Additionally, we defined 121 patients with other neurological diseases who did not have peripheral neuropathy as disease controls. The SNAP amplitudes were compared between the two groups. We also explored the relationship between SNAP amplitudes and FND-specific clinical symptoms in patients with FND.

**Results:**

No differences were observed in SNAP amplitudes adjusted for age between patients with FND who had sensory manifestations and disease control patients. Additionally, no differences were observed between patients with FND who had and did not have FND-specific clinical symptoms.

**Conclusion:**

The SNAP amplitude in patients with FND who had sensory manifestations was equivalent to that in controls.

## 1. Introduction

Functional neurological disorders (FNDs) are characterized by abnormal motor and sensory manifestations that are not observed in other medical conditions. FND, also known as hysteria or a conversion disorder, affects ~15% of outpatients in neurological clinics (1, 2). Patients with FND may experience sensory symptoms and weakness. However, clinical history and examination do not always suggest organic disease.

The study of sensory manifestations in FND reported variations in sensitivity and specificity (3). Non-anatomical sensory loss, inconsistency, and non-reproducibility of sensory signs revealed high sensitivity and specificity, but symptoms were inaccurately defined (4). Recently, functional magnetic resonance imaging has been used to investigate FND. Functional movement disorder suggests amygdala hyper-activation as a potential biomarker (5), and the presence of unilateral sensory conversion disorder suggests abnormal activity in the right limbic cortex and temporoparietal junction (6). Moreover, from the perspective of hypothalamic pituitary adrenal axis dysfunction (7), salivary cortisol may be a potential biomarker of functional movement disorder, but this is controversial (8, 9).

Although FND is thought to have a common brain basis of top–down prediction errors and impairments of bottom–up sensory input integration (10, 11), the contribution of peripheral sensory input has not been investigated in detail. FND with somatosensory manifestation is better suited for investigation than other special sensory FND because peripheral nerve function can be examined directly.

In daily clinical practice, nerve conduction studies are useful in FND diagnosis with sensory manifestations because no abnormalities are discovered in the symptomatic limb, unlike organic neuropathy. However, whether the features of nerve function in patients with FND are identical to normal remains unknown.

We occasionally encounter high sensory nerve action potential (SNAP) amplitudes in patients with FND who have sensory manifestations. Tipton et al. (12) retrospectively investigated 400 consecutive patients with normal nerve conduction and discovered that patients with multifocal sensory symptoms had higher SNAP (“supranormal” SNAP) amplitudes. They speculated that supranormal SNAP may be an indicator of nerve hyper-excitability. Thus, we hypothesized that some FND patients with sensory manifestations may reveal SNAP with relatively high amplitudes compared to the general population.

Therefore, we aimed to investigate the association between SNAP amplitudes and FND with sensory manifestations. Here, we compared SNAP amplitudes (1) between patients with FND who had sensory manifestations and disease control patients and (2) between patients with FND with and without FND-specific clinical symptoms.

## 2. Methods

### 2.1. Study population

We designed a retrospective study that included patients with FND who complained of sensory symptoms at Kobe University's neurologic clinic between April 2014 and March 2019. The diagnosis of FND was based on the diagnostic criteria outlined in DSM-5 (13). The exclusion criterion was patients with a background of peripheral neuropathy, including history of drug or alcohol abuse, vitamin B1 or B12 deficiency, diabetes, Sjögren syndrome, or systemic lupus erythematosus. Finally, we included 76 patients with FND and evaluated their SNAP amplitudes. Furthermore, we recruited 121 patients as disease controls who were diagnosed with myelopathy or other neurological diseases in our hospital, excluding any peripheral neuropathies and were reported to have normal nerve conduction studies. The Kobe University Ethics Committee approved our study (approval number: B210160).

### 2.2. Definition of specific clinical symptoms in patients with FND who have sensory manifestations

In this study, we defined three FND-specific clinical symptoms in patients with FND who had sensory manifestations. First, an “unexplained motor symptom” is a concomitant motor symptom that cannot be explained using neurological examinations. Second, an “unexplained sensory distribution symptom” is a bizarre distribution that cannot be explained using a single anatomical lesion. Third, a “non-persistent sensory symptom” is a somatosensory complaint that fluctuates without a trigger or treatment.

### 2.3. Nerve conduction study

We performed nerve conduction studies using an electromyograph (MEB-2300, Nihon Kohden, Tokyo, Japan) on outpatients under the supervision of board-certified clinical neurophysiologists or skilled neurologists. The studies were performed under proper skin temperatures of over 32°C and 31°C on the upper and lower limbs, respectively. We measured some or all of the median, ulnar, and sural nerves, unilaterally or bilaterally. All SNAPs were recorded using surface electrodes, and sensory nerve stimulation was performed antidromically. The active electrode of the median nerve was placed on the proximal interphalangeal joint of the index finger, the ulnar nerve was placed on the proximal interphalangeal joint of the small finger and that of the sural nerves was placed between the outer ankle and heel. Each reference electrode was located 3 cm distal to the active electrode. The site of electrical stimulation was 2 cm proximal to the distal crease of the wrist at the median and ulnar nerves and 14 cm proximal to the recording electrode at the sural nerve. SNAP amplitudes were defined from the baseline to the negative peak.

### 2.4. Study design

**Figure 1:**
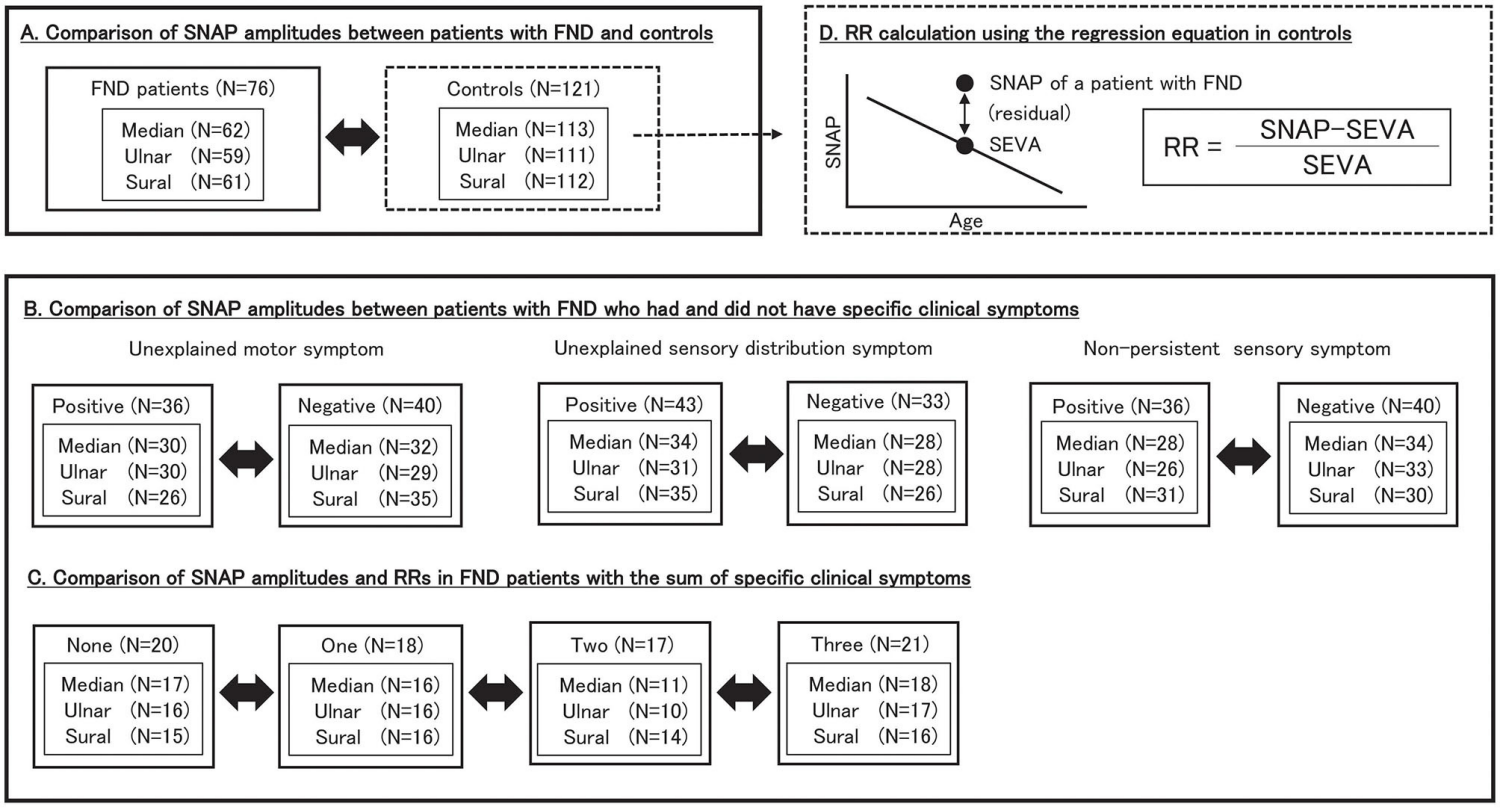
Study design. We included 76 patients with FND and 121 controls. **(A)** We compared SNAP amplitudes between patients with FND and controls. **(B)** We compared SNAP amplitudes between patients with FND who had and did not have specific clinical symptoms. **(C)** We compared SNAP amplitudes and RRs in FND patients with the number of specific clinical symptoms. **(D)** RR was calculated as follows: RR = (SNAP–SEVA)/SEVA. SEVA was calculated for each patient with FND using the regression equation for age and SNAP in controls. SNAP, sensory nerve action potential; FND, functional neurological disorder; RR, residual rate; SEVA, SNAP estimate value for age.

Figure 1 illustrates the study design, number of patients, and SNAP values for each group. In patients with FND, SNAP amplitudes were defined as the value on the symptomatic limb or the average value if the tests were performed bilaterally. In the control groups, SNAP amplitudes were defined as the value if unilateral and the average value if bilateral. We compared SNAP adjusted for age between patients with FND and controls. Furthermore, we compared SNAP adjusted for age in patients with FND who had and did not have specific clinical symptoms to elucidate characteristics in patients who displayed substantial functional symptoms within the FND group. Moreover, SNAP was adjusted for age in FND patients with the number of specific clinical symptoms. In addition, we calculated the SNAP estimated value for each age (SEVA) using the regression equation for age and SNAP in the control group. We defined the residual rate (RR) as follows:


RR= (each SNAP amplitude  SEVA)/SEVA

RR was calculated because SNAP is affected by aging, and differences in age distribution may occur among the study groups. Therefore, we believe that RR analysis reduces the effect of aging on SNAP.

### 2.5. Statistical analysis

In terms of demographics, age, body mass index (BMI), and SNAP amplitudes were analyzed using a *t*-test between two groups and analysis of variance (ANOVA) among the four groups. Sex was analyzed using Pearson's chi-square test. SNAP amplitudes between patients with FND and controls, between patients with FND who had and did not have specific clinical symptoms, and among patients with FND with the number of specific clinical symptoms were compared using the analysis of covariance (ANCOVA) adjusted for age. RR among patients with FND with the number of specific clinical symptoms was compared using ANOVA. All statistical analyses were performed using EZR, a graphical user interface for R (14).

## 3. Results

### 3.1. Comparison of SNAP amplitudes between patients with FND and controls

**Figure 2:**
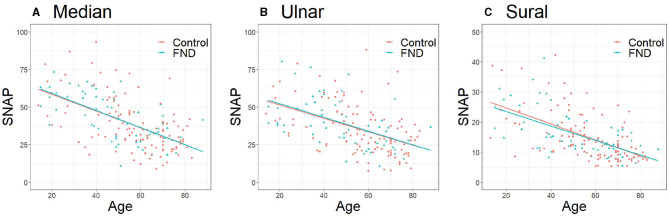
Comparison of SNAP between patients with FND and controls. **(A)** Median (*P* = 0.94, regression equation; DC y = −0.56 x +69.5, FND y = −0.65 x +73.1), **(B)** Ulnar (*P* = 0.84, regression equation; DC: y = −0.45 x +60.3, FND y = −0.54 x +64.7), **(C)** Sural (*P* = 0.72, regression equation; DC: y = −0.27 x +30.0, FND: y = −0.24 x +28.5). Data were analyzed using analysis of covariance adjusted for age. SNAP, sensory nerve action potential; FND, functional neurological disorder; DC, disease control.

Table 1 presents the clinical characteristics of patients with FND and controls. Patients with FND were younger than controls (Median, Ulnar: *P* < 0.01, Sural: *P* = 0.03), and no differences were observed in sex and BMI between the two groups. No significant differences were observed in SNAP amplitudes of each of the three nerves between patients with FND and controls (median: *P* = 0.09, ulnar: *P* = 0.12, and sural: *P* = 0.34). Three regression equations for age and SNAP in controls were calculated (median: y = −0.56 x +69.5, ulnar: y = −0.45 x +60.3, sural: y = −0.27 x +30.0). No significant difference was observed in the SNAP amplitudes of each of the three nerves using ANCOVA adjusted for age between patients with FND and controls (Figure 2).

**Table 1:** Clinical characteristics of patients with FND and controls.

	**Median**			**Ulnar**			**Sural**		
	**Control (*****N*** = **113)**	**FND (*****N*** = **62)**	***P*** **-value**	**Control (*****N*** = **111)**	**FND (*****N*** = **59)**	***P*** **-value**	**Control (*****N*** = **112)**	**FND (*****N*** = **61)**	***P*** **-value**
Age, mean (SD), year	56.6 (17.1)	48.8 (17.9)	< 0.01	56.6 (17.2)	48.4 (18.1)	< 0.01	56.0 (17.5)	49.9 (18.6)	0.03
Sex (female), *N* (%)	60 (53)	33 (53)	1.00	59 (53)	33 (56)	0.85	60 (54)	38 (61)	0.39
BMI, mean (SD), kg/m^2^	22.5 (4.0)	23.0 (2.6)	0.70	22.5 (4.1)	22.9 (2.6)	0.70	22.4 (3.8)	22.7 (2.1)	0.77
SNAP amplitude, mean (SD), μV	38.1 (17.5)	42.7 (15.7)	0.09	35.1 (16.7)	39.3 (16.0)	0.12	15.1 (8.1)	16.3 (7.5)	0.34

BMI (Median. DC: N = 84, FND: N = 19, Ulnar. DC: N = 84, FND: N = 18, Sural. DC: N = 83, FND: N = 18). Age, BMI, and SNAP amplitudes were analyzed using the t-test, and sex was analyzed using Pearson's chi-square test. DC, disease control; FND, functional neurological disorder; BMI, body mass index; SNAP, sensory nerve action potential; SD, standard deviation.

### 3.2. Comparison of SNAP amplitudes between patients with FND who had and did not have specific clinical symptoms

**Figure 3:**
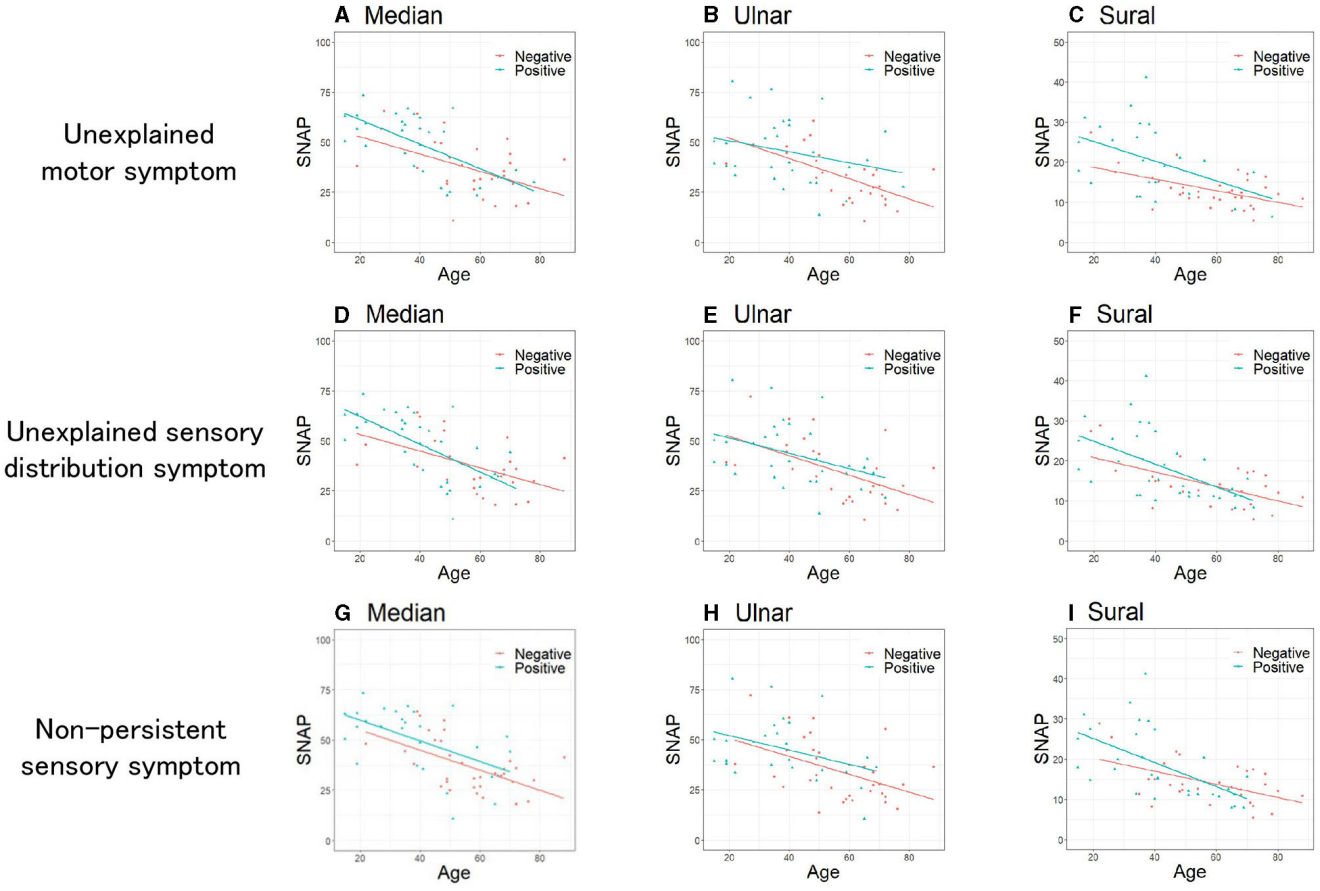
Comparison of SNAP between patients with FND with and without specific clinical symptoms. Unexplained motor symptoms **(A–C)**. **(A)** Median (*P* = 0.34), **(B)** Ulnar (*P* = 0.18), **(C)** Sural (*P* = 0.04). Unexplained sensory distribution symptoms **(D–F)**. **(D)** Median (*P* = 0.83), **(E)** Ulnar (*P* = 0.59), **(F)** Sural (*P* = 0.63). Non-persistent sensory symptoms **(G–I)**. **(G)** Median (*P* = 0.21), **(H)** Ulnar (*P* = 0.36), **(I)** Sural (*P* = 0.66). Data were analyzed using the analysis of covariance adjusted for age. SNAP, sensory nerve action potential; FND, functional neurological disorder.

To elucidate patient characteristics with more substantial functional symptoms within the FND group, we divided the patients with FND with or without each of the three specific clinical symptoms (“unexplained motor symptom,” “unexplained sensory distribution symptom,” and “non-persistent sensory symptom”) and compared the SNAP of each of the three nerves between the two groups. Table 2 presents the clinical characteristics of patients with FND, with and without specific clinical symptoms. Patients with FND who experienced specific clinical symptoms were younger than those who did not (median, ulnar, and sural: *P* < 0.01). No sex differences were observed between the two groups. Patients with FND who experienced “non-persistent sensory symptom” had a higher BMI than patients who did not (median: *P* = 0.04, ulnar *P* = 0.03) despite the small sample size. Patients with FND who had “unexplained motor symptom”, “unexplained sensory distribution symptom”, and “non-persistent sensory symptom” had a larger SNAP amplitude than those who did not (“unexplained motor symptom”; median, ulnar, sural: *P* < 0.01, “unexplained sensory distribution symptom”; median: *P* = 0.04, sural: *P* = 0.04, “non-persistent sensory symptom”; median, ulnar: *P* < 0.01, sural: *P* = 0.03). Patients with FND who had individual clinical symptoms revealed that SNAP amplitudes of the sural nerve using ANCOVA adjusted for age in the group with “unexplained motor symptom” were significantly larger than those without (Figure 3, *P* = 0.04). However, the age distribution between the two groups differed.

**Table 2:** Clinical characteristics of patients with FND who had and did not have specific clinical symptoms.

**A. Unexplained motor symptom**									
	**Median**			**Ulnar**			**Sural**		
	**NEG (*****N*** = **32)**	**POS (*****N*** = **30)**	***P*** **-value**	**NEG (*****N*** = **29)**	**POS (*****N*** = **30)**	***P*** **-value**	**NEG (*****N*** = **35)**	**POS (*****N*** = **26)**	***P*** **-value**
Age, mean (SD), year	57.1 (15.4)	40.0 (16.3)	< 0.01	57.6 (15.0)	39.4 (16.4)	< 0.01	58.2 (15.8)	48.4 (18.3)	< 0.01
Sex (female), *N* (%)	16 (50)	17 (57)	0.79	15 (52)	18 (60)	0.71	21 (60)	17 (65)	0.87
BMI, mean (SD), kg/m^2^	22.9 (2.0)	23.1 (2.9)	0.99	22.9 (2.0)	22.9 (3.0)	0.98	22.6 (2.0)	22.7 (2.3)	0.94
SNAP amplitude, mean (SD), μV	36.7 (13.8)	49.0 (15.3)	< 0.01	33.0 (12.5)	45.4 (16.7)	< 0.01	13.2 (4.4)	20.5 (8.8)	< 0.01
**B. Unexplained sensory distribution symptom**									
	**Median**			**Ulnar**			**Sural**		
	**NEG (*****N*** = **28)**	**POS (*****N*** = **34)**	***P*** **-value**	**NEG (*****N*** = **28)**	**POS (*****N*** = **31)**	***P*** **-value**	**NEG (*****N*** = **26)**	**POS (*****N*** = **35)**	***P*** **-value**
Age, mean (SD), year	56.1 (17.2)	42.7 (16.4)	< 0.01	56.0 (17.4)	41.9 (16.4)	< 0.01	57.5 (18.9)	44.3 (16.4)	< 0.01
Sex (female), *N* (%)	14 (50)	19 (56)	0.65	15 (54)	18 (58)	0.93	16 (62)	22 (63)	1.00
BMI, mean (SD), kg/m^2^	22.2 (2.3)	24.2 (2.6)	0.09	22.2 (2.3)	24.1 (2.7)	0.14	22.3 (2.2)	23.0 (2.1)	0.57
SNAP amplitude, mean (SD), μV	38.2 (13.5)	46.4 (16.6)	0.04	35.1 (15.5)	43.1 (15.6)	0.053	14.1 (5.8)	18.0 (8.2)	0.04
**C. Non-persistent sensory symptom**									
	**Median**			**Ulnar**			**Sural**		
	**NEG (*****N*** = **34)**	**POS (*****N*** = **28)**	***P*** **-value**	**NEG (*****N*** = **33)**	**POS (*****N*** = **26)**	***P*** **-value**	**NEG (*****N*** = **30)**	**POS (*****N*** = **31)**	***P*** **-value**
Age, mean (SD), year	56.7 (14.4)	39.2 (14.4)	< 0.01	56.2 (15.4)	38.5 (16.6)	< 0.01	57.5 (17.1)	42.5 (17.1)	< 0.01
Sex (female), *N* (%)	18 (53)	15 (54)	1.00	19 (58)	14 (54)	0.98	19 (63)	19 (61)	1.00
BMI, mean (SD), kg/m^2^	22.1 (2.2)	24.5 (2.4)	0.04	21.9 (2.2)	24.5 (2.4)	0.03	22.3 (2.1)	23.1 (2.2)	0.51
SNAP, amplitude, mean (SD), μV	36.7 (12.7)	50.0 (16.1)	< 0.01	34.4 (14.6)	45.4 (15.8)	< 0.01	14.1 (5.4)	18.4 (8.7)	0.03

BMI **(A)** Unexplained positive motor symptoms (Median. Negative: N = 8; Positive: N = 11; Ulnar. Negative: N = 8; Positive: N = 10; Sural. Negative: N = 9, Positive: N = 9); **(B)** Unexplained sensory distribution symptoms (Median. Negative: N = 11; Positive: N = 8; Ulnar. Negative: N = 11; Positive: N = 7; Sural. Negative: N = 9, Positive: N = 9); **(C)** Non-persistent sensory symptoms (Median. Negative: N = 12; Positive: N = 7; Ulnar. Negative: N = 11; Positive: N = 7; Sural. Negative: N = 11; positive: N = 7). Age, BMI, and SNAP amplitudes were analyzed using the t-test, and sex was analyzed using Pearson's chi-square test. FND, functional neurological disorder; BMI, body mass index; SNAP, sensory nerve action potential; NEG, negative; POS, positive.

### 3.3. Comparison of SNAP amplitudes and RRs in FND patients with the number of specific clinical symptoms

**Figure 4:**
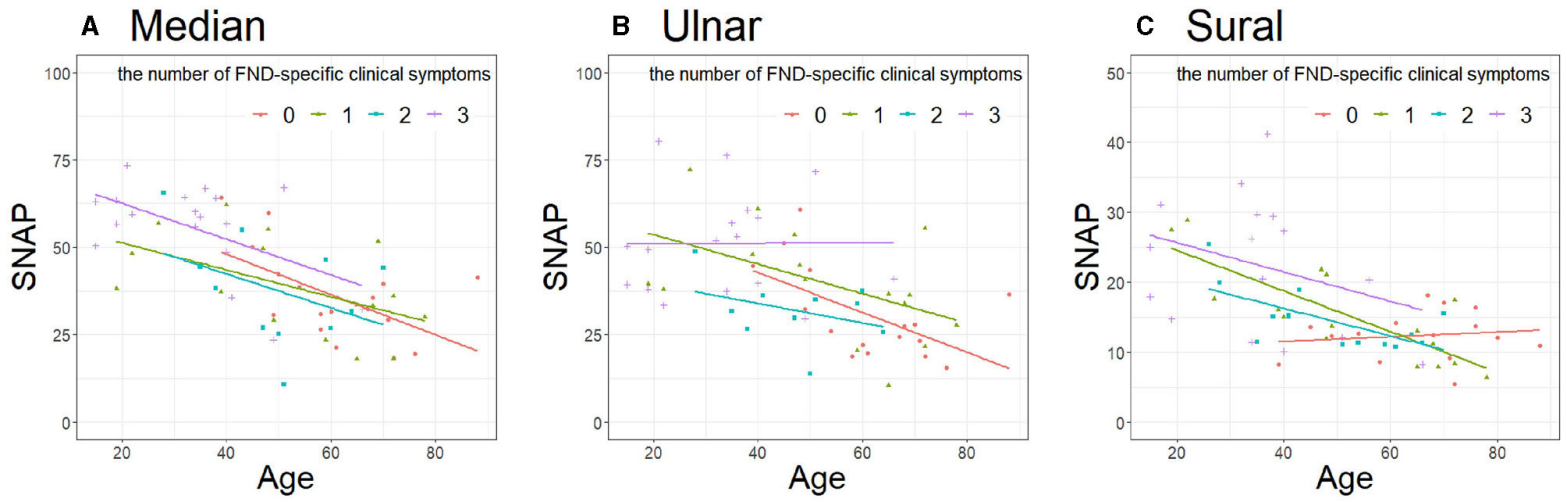
Comparison of SNAP in patients with FND with the number of specific clinical symptoms. **(A)** Median (*P* = 0.17), **(B)** Ulnar (*P* = 0.049), **(C)** Sural (*P* = 0.13). Data were analyzed using analysis of covariance adjusted for age. SNAP, sensory nerve action potential; FND, functional neurological disorder.

Considering the influence of age difference between the two FND subgroups, we performed a sub-analysis using the RR. Furthermore, we considered the extent of the functional symptoms by summing the symptoms. Table 3 presents the clinical characteristics of the patients with FND with the number of specific clinical symptoms. Patients with FND who experienced more specific clinical symptoms were younger than controls (median, ulnar, and sural: *P* < 0.01). No differences in sex and BMI were observed among the four groups. SNAP amplitudes of each of the three nerves in patients with FND, with more specific clinical symptoms, were larger (median, ulnar, and sural: *P* < 0.01). SNAP amplitudes of the ulnar nerve using ANCOVA adjusted for age in the group with the number of specific clinical symptoms were significantly larger than those without (Figure 4, *P* = 0.049). However, the RRs of all three nerves did not differ among the four groups (Table 4).

**Table 3:** Clinical characteristics of patients with FND with the number of specific clinical symptoms.

**Median**	**None (*N* = 17)**	**One (*N* = 16)**	**Two (*N* = 11)**	**Three (*N* = 18)**	***P*-value**
Age, mean (SD), year	60.8 (12.7)	52.4 (18.9)	49.6 (13.0)	33.7 (13.7)	< 0.01
Sex (female), *N* (%)	8 (47)	9 (56)	6 (55)	10 (56)	0.95
BMI, mean (SD), kg/m^2^	23.0 (2.1)	21.2 (2.0)	24.9 (0.0)	24.6 (2.6)	0.10
SNAP amplitude, mean (SD), μV	36.0 (12.9)	38.7 (13.9)	37.7 (15.5)	55.0 (13.1)	< 0.01
**Ulnar**	**None (*****N*** = **16)**	**One (*****N*** = **16)**	**Two (*****N*** = **10)**	**Three (*****N*** = **17)**	***P*** **-value**
Age, mean (SD), year	61.0 (13.1)	52.4 (18.9)	47.3 (11.8)	33.3 (13.9)	< 0.01
Sex (female), *N* (%)	8 (50)	9 (56)	7 (70)	9 (53)	0.78
BMI, mean (SD), kg/m^2^	23.0 (2.1)	21.2 (2.0)	NA	24.6 (2.6)	0.06
SNAP amplitude, mean (SD), μV	30.8 (13.1)	40.0 (15.9)	31.8 (9.1)	51.0 (15.0)	< 0.01
**Sural**	**None (*****N*** = **15)**	**One (*****N*** = **16)**	**Two (*****N*** = **14)**	**Three (*****N*** = **16)**	***P*** **-value**
Age, mean (SD), year	64.9 (13.7)	51.8 (18.9)	48.4 (14.3)	35.3 (14.4)	< 0.01
Sex (female), *N* (%)	9 (60)	10 (63)	9 (64)	10 (63)	0.88
BMI, mean (SD), kg/m^2^	22.9 (2.1)	21.9 (2.0)	22.5 (3.5)	23.4 (2.1)	0.68
SNAP amplitude, mean (SD), μV	12.3 (3.5)	15.3 (6.8)	14.6 (4.3)	22.5 (9.6)	< 0.01

BMI (Median. None: N = 6, One: N = 6, Two: N = 1, Three: N = 6, Ulnar. None: N = 6, One: N = 6, Two: N = 0, Three: N = 6, Sural. None: N = 5, One: N = 6, Two: N = 2, Three: N = 5). Age, BMI, and SNAP amplitudes were analyzed using analysis of variance, and sex was analyzed using Pearson's chi-square test. FND, functional neurological disorder; BMI, body mass index; SNAP, sensory nerve action potential; SD, standard deviation.

**Table 4:** Residual rates of each of the three nerves in patients with FND with the number of specific clinical symptoms.

	**None (*N* = 20)**	**One (*N* = 18)**	**Two (*N* = 17)**	**Three (*N* = 21)**	***P*-value**
Median, mean (SD)	0.01 (0.35)	−0.03 (0.33)	−0.09 (0.36)	0.10 (0.24)	0.43
Ulnar, mean (SD)	−0.06 (0.37)	0.10 (0.39)	−0.18 (0.21)	0.14 (0.37)	0.09
Sural, mean (SD)	0.07 (0.46)	−0.05 (0.29)	−0.12 (0.20)	0.10 (0.46)	0.33

Median: None: N = 17, One: N = 16, Two: N = 11, Three: N = 18, Ulnar: None: N = 16, One: N = 16, Two: N = 10, Three: N = 17, Sural: None: N = 15, One: N = 16, Two: N = 14, Three: N = 16. The residual rates were analyzed using analysis of variance. FND, functional neurological disorder; SD, standard deviation.

## 4. Discussions

To the best of our knowledge, this is the first study to examine SNAP amplitudes in patients with FND. We compared SNAP amplitudes (1) between patients with FND who had sensory manifestations and controls and (2) between patients with FND with and without FND-specific clinical symptoms. However, no difference was observed. This may be because in-house normative data of SNAP amplitudes in hospitals or laboratories may include the data of patients with FND. Therefore, we cannot consider the data of SNAP amplitudes of patients with FND as normative data; however, we reported that there were no significant differences between the FND and the control data.

Based on our clinical experience and the report by Tipton et al. (12) we hypothesized that patients with FND who had sensory manifestations had higher SNAP amplitudes than the normal population. However, no significant amplitude differences were observed when the SNAP amplitudes and those adjusted for age were compared between patients with FND with sensory manifestations and controls. The clinical impression of larger SNAP amplitudes in patients with FND could be due to differences in the age distribution of FND patients. Moreover, patients in the FND group displayed various functional symptoms. Therefore, it is essential to identify patients with FND with high purity to elucidate the substantial characteristics of patients with FND who have sensory manifestations. Sub-analyses for individual clinical symptoms revealed that patients with FND with only one subcategory (“unexplained motor symptom,” Sural: *P* = 0.04) had significantly higher SNAP amplitudes adjusted for age, resulting in the effect of age distribution. We compared SNAP amplitudes adjusted for age in patients with FND with the number of specific clinical symptoms; the results showed differences between the groups (ulnar: *P* = 0.049). Additionally, we compared the RRs among patients with FND with the number of specific clinical symptoms to reduce the effect of aging on SNAP. Yet there were no significant differences between the groups. Patients with FND consisted of heterogeneous groups; however, in this study, no clear correlation between patients with FND and SNAP amplitudes was observed.

Sensory nerve hyper-excitability using threshold tracking is useful for assessing neuropathic pain caused by peripheral neuropathy (15). However, a recent multicenter observational study discovered that axonal excitability did not differ with or without pain in patients with diabetic and chemotherapy-induced polyneuropathy (16). Nonetheless, sensory nerve hyper-excitability in patients with sensory manifestations without peripheral neuropathy, such as FND, has not been elucidated. SNAP amplitudes decreased with age, but sensory nerve hyper-excitability in healthy controls using threshold tracking (QTRAC program) did not change with age (17). Therefore, if FND affects the peripheral and central nervous systems, measuring sensory nerve hyper-excitability using threshold tracking in patients with FND who have sensory manifestations may be useful for pathophysiological evaluation. In addition, the SNAP amplitude is highly dependent on single action potential durations and their temporal synchronization. Thus, in future, if patients with FND manifesting sensory symptoms have a large amplitude of SNAP beyond the age effect, action potential duration and synchronization should be considered.

Nonetheless, this study had some limitations. First, we recruited patients with disease as controls rather than healthy controls. Second, we defined exclusion criteria to exclude patients with organic peripheral neuropathy as the cause but were unable to exclude a patient with neurological disease co-existing with FND (18). Furthermore, myelopathy often causes a lower SNAP amplitude (19), but its existence was not thoroughly examined in our retrospective cohort. The number of patients with recorded BMI was small though BMI affects SNAP amplitudes (20). However, BMI showed no difference between groups as possible numbers. Finally, we were unable to evaluate organic small-fiber neuropathy using the current sensory conduction study. Thus, some patients may have reduced SNAP amplitudes due to co-existing small-fiber neuropathy.

## 5. Conclusion

SNAP amplitudes of patients with FND with sensory manifestations did not differ from those of controls. Patients with FND with higher diagnostic certainty displayed FND-specific symptoms and did not reveal unexplainable higher SNAP amplitudes. Thus, the similarity of SNAP amplitudes between patients with FND and controls suggests no correlation between FND and SNAP amplitudes.

## Data availability statement

The original contributions presented in the study are included in the article/supplementary material, further inquiries can be directed to the corresponding author.

## Ethics statement

The studies involving humans were approved by the Kobe University Ethics Committee. The studies were conducted in accordance with the local legislation and institutional requirements. Written informed consent for participation was not required from the participants or the participants' legal guardians/next of kin because this study is a retrospective study using clinical information in the opt-out approach.

## Author contributions

KM: Data curation, Formal analysis, Investigation, Methodology, Project administration, Writing–original draft. KS: Conceptualization, Formal analysis, Investigation, Methodology, Project administration, Writing–review and editing. RM: Formal analysis, Supervision, Writing–review and editing.
