# Corrigendum: Sensory nerve action potential in patients with functional neurological disorders with sensory manifestations

**DOI:** 10.3389/fneur.2024.1374287

**Published:** 2024-02-09

**Authors:** Kohei Morimoto, Kenji Sekiguchi, Riki Matsumoto

**Affiliations:** Division of Neurology, Kobe University Graduate School of Medicine, Kobe, Japan

**Keywords:** functional neurological disorder, sensory symptom, nerve conduction study, sensory nerve action potential (SNAP), FND

In the published article, there was an error in the Funding statement. The funding from the Ministry of Health, Labor and Welfare was mistakenly omitted. The correct Funding statement appears below.

